# Structure Learning of Bayesian Network Based on Adaptive Thresholding

**DOI:** 10.3390/e21070665

**Published:** 2019-07-08

**Authors:** Yang Zhang, Limin Wang, Zhiyi Duan, Minghui Sun

**Affiliations:** 1 College of Computer Science and Technology, Jilin University, Changchun 130012, China; 2 Key Laboratory of Symbolic Computation and Knowledge Engineering of Ministry of Education, Jilin University, Changchun 130012, China

**Keywords:** Bayesian network classifiers, mutual information, conditional mutual information, thresholding

## Abstract

Direct dependencies and conditional dependencies in restricted Bayesian network classifiers (BNCs) are two basic kinds of dependencies. Traditional approaches, such as filter and wrapper, have proved to be beneficial to identify non-significant dependencies one by one, whereas the high computational overheads make them inefficient especially for those BNCs with high structural complexity. Study of the distributions of information-theoretic measures provides a feasible approach to identifying non-significant dependencies in batch that may help increase the structure reliability and avoid overfitting. In this paper, we investigate two extensions to the *k*-dependence Bayesian classifier, MI-based feature selection, and CMI-based dependence selection. These two techniques apply a novel adaptive thresholding method to filter out redundancy and can work jointly. Experimental results on 30 datasets from the UCI machine learning repository demonstrate that adaptive thresholds can help distinguish between dependencies and independencies and the proposed algorithm achieves competitive classification performance compared to several state-of-the-art BNCs in terms of 0–1 loss, root mean squared error, bias, and variance.

## 1. Introduction

Classification is one of the most important tasks in machine learning. The basic problem of supervised classification is the induction of a model with feature set $X=\{X_{1},\cdots ,X_{n}\}$ that classifies testing instance (example) $x=\{x_{1},\cdots ,x_{n}\}$ into one of the several class labels $\left\{c_{1},\cdots ,c_{m}\right\}$ of class variable *C*. Bayesian network classifiers (BNCs) have many desirable properties over other numerous classification models, such as model interpretability, the ease of implementation, the ability to deal with multi-class classification problems and the comparable classification performance [1]. A BNC or *B* assigns the most probable label with the maximum posterior probability to **x** by calculating the posterior probability for each class label that is:

(1) $$\begin{array}{cc} \arg\max_{C}P_{B}\left(c|x\right) & =\arg\max_{C}\frac{P_{B}\left(x,c\right)}{P_{B}\left(x\right)}\propto \arg\max_{C}P_{B}\left(x,c\right), \end{array}$$

where class label $c\in \{c_{1},\cdots ,c_{m}\}$.

**Figure 1:**
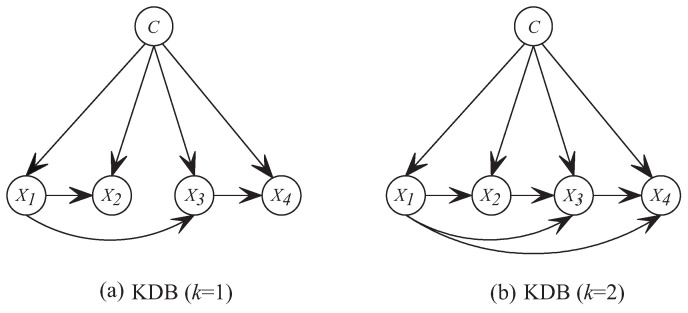
Examples of network structures with four features for KDB.

Although unrestricted BNCs are the least biased, the search-space that is needed to train such a model increases exponentially with the number of features [2]. The arising complexity issues limit the study of unrestricted BNCs and it has led to the study of restricted BNCs, from 0-dependence naive Bayes (NB) [3,4,5], 1-dependence tree-augmented naive Bayes (TAN) [6] to *k*-dependence Bayesian classifier (KDB) [7]. These classifiers take class variable as the common parent of all predictive features and use different learning strategies to explore the conditional dependence among features. KDB has numerous desirable characteristics in structure learning. For example, it has satisfactory classification accuracy while dealing with large quantities of data [2]. In addition, KDB uses a single parameter, *k*, to set the maximum number of parents for any feature and thus controls the structure complexity. KDB first determines the feature order by comparing MI. Suppose that the order is $\left\{X_{1},\cdots ,X_{n}\right\}$, then $X_{i}$ can select at most *k*, or more precisely min{i−1,k}, features as parents from its candidates $\left\{X_{1},\cdots ,X_{i-1}\right\}$. These parents correspond to the min{i−1,k} largest CMI values. Figure 1 shows two examples, i.e., K${}_{1}$DB (KDB with k=1) and K${}_{2}$DB (KDB with k=2). Suppose that $I\left(X_{1};C\right)>I\left(X_{2};C\right)>I\left(X_{3};C\right)>I\left(X_{4};C\right)$, then the feature order is $\left\{X_{1},X_{2},X_{3},X_{4}\right\}$. If $I\left(X_{3};X_{4}|C\right)>I\left(X_{1};X_{4}|C\right)>I\left(X_{2};X_{4}|C\right)$, $X_{4}$ in K${}_{1}$DB chooses $X_{3}$ as its only parent and $X_{4}$ in K${}_{2}$DB chooses $\left\{X_{1},X_{3}\right\}$ as its parents from candidates $\left\{X_{1},X_{2},X_{3}\right\}$.

There are two basic kinds of dependencies in restricted BNCs: (1) direct dependence between feature $X_{i}$ and *C* that can be quantified by mutual information (MI) $I(X_{i};C)$, and (2) conditional dependence between $X_{i}$ and $X_{j}$ given *C* that can be measured by conditional mutual information (CMI) $I(X_{i};X_{j}|C)$. Many researchers have exploited methods, such as filter and wrapper [8,9,10,11,12,13], to select direct dependencies by removing redundant features. The filter approach operates independently of any learning algorithms that rank the features by some criteria and omit all features that do not achieve a sufficient score [14,15,16]. The wrapper approach evaluates the feature subsets every time and may produce better results. For example, Backwards Sequential Elimination (BSE) [17] uses a simple heuristic wrapper approach that seeks a subset of the available features that minimizes 0–1 loss on the training set. Forward Sequential Selection (FSS) [18] uses the reverse search direction to BSE. Although the filter and wrapper approaches have proved to be beneficial in domains with highly correlated features, the learning procedure ends only when there is no accuracy improvement, thus they are expensive to run and can break down with very large numbers of features [8,19,20]. Suppose that we need to select *m* from *n* features for classification, BSE or FSS will construct $P_{n}^{m}$ or $\frac{n!}{m!}$ candidate BNCs to judge if there exist non-significant features or direct dependencies. It is even more difficult for BSE or FSS to select the conditional dependencies. For example, the network topology of KDB consists of $nk-\frac{k^{2}}{2}-\frac{k}{2}$ conditional dependencies [21]. If BSE or FSS evaluate them one by one to identify those relatively non-significant ones, the high computational overheads is almost unbearable and few approaches are proposed to address this issue.

**Figure 2:**
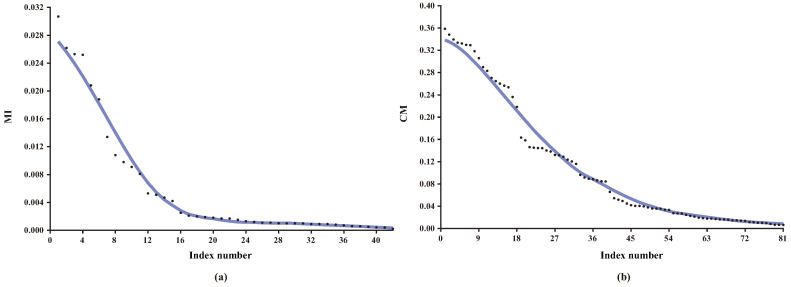
The distributions of (**a**) MI; (**b**) CMI values on dataset Connect-4. Note that the MI and CMI values are sorted in descending order.

Obviously, how to efficiently identify non-significant direct and conditional dependencies are two key issues to learn BNC. Strictly speaking, there exist no direct or conditional independence due to the fact that the MI and CMI values are non-negative. However, weak dependencies, if introduced into the network topology, will result in overfitting and classification bias. For KDB, all features are indiscriminately conditionally dependent on at most *k* parent features even if the conditional dependencies are very weak. Discarding these redundant features or weak conditional dependencies can help increase structure reliability and avoid overfitting. Figure 2 presents the distributions of MI and CMI values for K${}_{2}$DB (KDB with *k* = 2) on dataset Connect-4, which has 67,557 instances (or examples), 42 features and three classes. As shown in Figure 2a, there exist minor differences among some MI values, thus the significance of corresponding direct dependencies is almost the same and they can be treated in batch. From Figure 2b, the same also applies to CMI and corresponding conditional dependencies.

The filter approaches have computational efficiency while the wrapper approaches may produce better results. The algorithm proposed combines the characteristics of filter with wrapper approaches to exploit the complementary strengths. In this paper, we propose to group the direct (or conditional) dependencies into different batches using adaptive thresholds. We assume that there exists no significant difference between the MI (or CMI) values in the same batch. Then, the basic idea of filter and wrapper will be applied to a select batch rather than single dependence for each iteration. This learning strategy can help achieve much higher efficiency compared to BSE (or FSS) while retaining competitive classification performance, and above all it provides a feasible solution for selecting conditional dependencies, the number of which increases exponentially as the number of features increases.

We investigate two extensions to KDB, MI-based feature selection and CMI-based dependence selection based on a novel adaptive thresholding method. The final BNC, Adaptive KDB (AKDB), evaluates the subsets of features and conditional dependencies using leave-one-out cross validation (LOOCV). In the remaining sections, we prove that applying feature selection and dependence selection techniques to KDB can alleviate the potential redundancy problem. We present extensive experimental results, which prove that AKDB significantly outperforms several other state-of-the-art BNCs in terms of 0–1 loss, root mean squared error (RMSE), bias and variance.

## 2. Restricted Bayesian Network Classifiers

For convenience, except for the algorithm names, all the used acronyms in this work are listed in Table 1. The structure of BNC can be described as a directed acyclic graph [22]. Nodes in structure represent the class variable *C* or features, edge $X_{i}\to X_{j}$ denotes probabilistic dependency relationship between these two features and $X_{i}$ is one of the immediate parent nodes of $X_{j}$. Thus, in a restricted BNC or *B*, class variable *C* is required as the common parent of all features and does not have any parents so the individual probability of *C* is P(c). We use $P_{B}\left(x_{i}|\pi_{i}\right)$ to denote the individual probability of feature $X_{i}$, where $\pi_{i}$ denotes the set of values of $X_{i}$’s parents. The joint probability distribution can be calculated as the product of $P_{B}\left(x_{i}|\pi_{i}\right)$ of all features and P(c) that is:

(2) $$P_{B}\left(x,c\right)=P\left(c\right)\prod_{i=1}^{n}P_{B}\left(x_{i}|\pi_{i}\right).$$

Unfortunately, the inference of an unrestricted BNC has been proved to be an NP-hard problem [23,24] and learning a restricted or pre-fixed BNC is one approach to deal with the intractable complexity. For example, NB [25,26] is the simplest classifier among restricted BNCs that assumes each feature is conditionally independent given the class variable *C*.

Since, in the real world, the dataset usually does not satisfy the independence assumption, this may cause a deterioration of the classification performance. KDB alleviates the independence assumption of NB that it constructs classifiers which allow feature $X_{i}$ within BNC to have at most *k* parent features. KDB firstly sets the feature order by comparing MI values and then calculates CMI values as the weights to measure the conditional relationship between features given *C* and select at most *k* parent features for one feature. MI and CMI are defined as follows:

(3) $$\left\{\begin{array}{c} I\left(X_{i};C\right)=\sum_{x_{i}\in X_{i}}\sum_{c\in C}P\left(x_{i},c\right)log_{2}\frac{P(x_{i},c)}{P\left(x_{i}\right)P\left(c\right)},\\ I\left(X_{i};X_{j}|C\right)=\sum_{x_{i}\in X_{i}}\sum_{x_{j}\in X_{j}}\sum_{c\in C}P\left(x_{i},x_{j},c\right)\log_{2}\frac{P(x_{i},x_{j}|c)}{P\left(x_{i}|c\right)P\left(x_{j}|c\right).} \end{array}\right.$$

For KDB, $I(X_{i};C)$ measures the direct dependence between $X_{i}$ and *C*. $I(X_{i};X_{j}|C)$ measures the conditional dependence between $X_{i}$ and $X_{j}$ given *C*. For a given training set with *n* features and the parameter *k*, KDB firstly calculates MI and CMI. Suppose that the feature order is $\left\{X_{1},\cdots ,X_{n}\right\}$ by comparing MI, $X_{i}$ will choose min(i−1,k) features with the highest CMI values from the first i−1 candidates. The structure learning procedure of KDB is depicted in Algorithm 1.

There have been some refinements that may improve KDB’s performance. Rodríguez and Lozano [27] proposed to extend KDB to a multi-dimensional classifier, which learned a population of classifiers (nondominated solutions) by a multi-objective optimization technique and the objective functions for the multi-objective approach are the multi-dimensional *k*-fold cross-validation estimations of the errors. Louzada [28] proposed to generate multiple KDB networks via a naive bagging procedure by obtaining the predicted values from the adjusted models, and then combine them into a single predictive classification.

**Algorithm 1:** Structure learning procedure of KDB: LearnStructure(T, L, *k*)

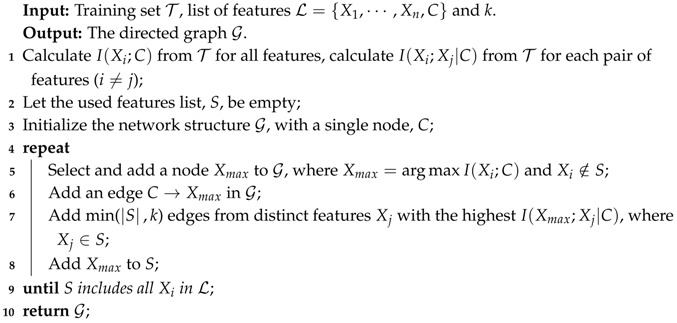



## 3. Adaptive KDB

MI and CMI are non-negative in Equation (3). $I(X_{i};C)=0$ (or $I(X_{i};X_{j}|C)=0$) if $X_{i}$ and *C* are independent (or $X_{i}$ and $X_{j}$ are conditionally independent given *C*). If $X_{i}$ and *C* are regarded as independent, the edge connecting them will be removed. Practically, the estimated MI is compared to a small threshold, in order to distinguish pairs of dependent and pairs of independent features [29,30,31,32]. In the following discussion, we mainly discuss how to choose the threshold of MI. The test for conditional independence using CMI is similar.

To refine the network structure, AKDB uses an adaptive threshold to filter out those non-significant dependencies. If the threshold is high, too many dependencies will be identified as non-significant and removed, and a sparse network may underfit the training data. In contrast, if the threshold is low, few dependencies will be identified as non-significant and a dense network may overfit the training data. The thresholds control AKDB’s bias-variance trade-off and, if appropriate thresholds are predefined, the lowest error will be achieved as this is a complex interplay between structure complexity and classification performance. Unfortunately, for different training datasets, the thresholds may differ and there are no formal methods to preselect the thresholds.

To guarantee satisfactory performance and overcome exhaustive experimentation, for KDB, given the feature order selected by KDB based on MI comparison, if feature $X_{i}$ is assumed to be independent of *C* when $I(X_{i};C)=0$, it will be at the end of the order and the edge $C\to X_{i}$ will be removed. Furthermore, $X_{i}$ may be dependent on other features, whereas no feature depends on it. That is, $X_{i}$ will be irrelevant to classification directly or indirectly. The problem of choosing the threshold of MI turns to choosing a feature subset. Many feature selection algorithms are based on forward selection or backwards elimination strategies [18,33]. They start with either an empty set of features or a full set of features, and then only one feature is added to BNC or removed from BNC for each iteration. Feature selection is a complex task that the search space for *n* features is $O(n^{2})$. Thus, it is impractical to search the whole space exhaustively, unless *n* is small. Our proposed algorithm, AKDB, extends KDB to adaptively select a threshold of MI and the threshold can help remove more than one feature at each step.

**Figure 3:**
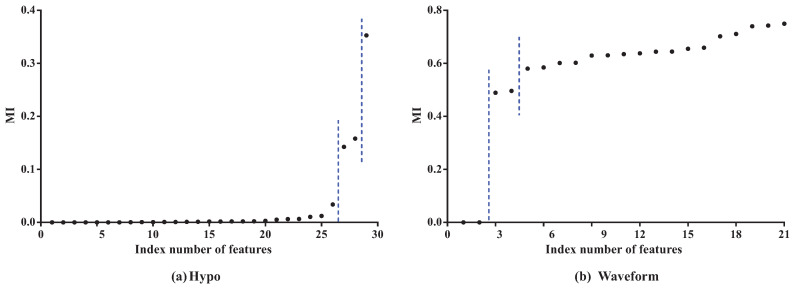
The MI values for K${}_{2}$DB on datasets Hypo and Waveform. Note that features are sorted in ascending order of $I(X_{i};C)$.

**Figure 4:**
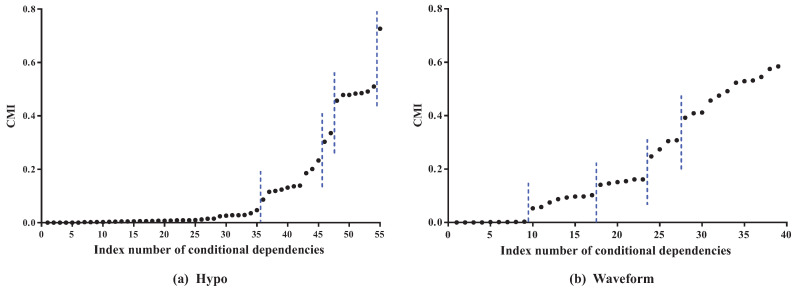
The CMI values for K${}_{2}$DB on datasets Hypo and Waveform. Note that the conditional dependencies are sorted in ascending order of $I(X_{i};X_{j}|C)$.

To clarify the basic idea, we take datasets Hypo and Waveform for a case study. Dataset Hypo has 3772 instances, 29 features and four classes. Dataset Waveform has 100,000 instances, 21 features and three classes. Corresponding MI values (see details in Table A1 and Table A2 in the Appendix A) and CMI values (see details in Table A3 and Table A4 in the Appendix A) for K${}_{2}$DB are, respectively, presented in Figure 3 and Figure 4.

As Figure 3 shows, the features can be divided into different parts according to the distribution of MI values. In dataset Hypo, we can see that the difference in MI values of the first 26 features is not obvious and that these features can be grouped into one part. The 27th and 28th features can be grouped into another part, and the 29th feature is the last part. On dataset Waveform, the features can also be divided into three parts. The distribution of CMI values is similar. As Figure 4 shows, the CMI values on datasets Hypo and Waveform are both divided into five groups. The difference in MI values in the same part should be non-significant and, if the MI values are small, corresponding features can be identified as redundant for classification and removed from BNC. The test for redundant conditional dependencies is similar. From Figure 3 and Figure 4, we can see that the thresholds for identifying redundancy differ greatly for different datasets. Thus, a threshold that maximizes a performance measure should be adapted to different datasets. $\hat{\mathrm{MI}}$ and $\hat{\mathrm{CMI}}$ are introduced as the adaptive thresholds of redundant features and redundant conditional dependencies, respectively. AMI and ACMI respectively denote the average MI and the average CMI, which are defined as follows, and are introduced in this paper as the benchmark thresholds to distinguish between strong and weak dependencies:

(4) $$\left\{\begin{array}{c} \mathrm{AMI}=\frac{1}{\left|X\right|}\sum_{X_{i}\in X}I\left(X_{i};C\right),\\ \mathrm{ACMI}=\frac{1}{\sum_{i=1}^{\left|F\right|}\left|\pi_{i}\right|}\sum_{X_{i}\in X}\sum_{X_{j}\in \pi_{i}}I\left(X_{i};X_{j}|C\right), \end{array}\right.$$

where $\left|\pi_{i}\right|$ is the size of $\pi_{i}$, $\left|X\right|$ and $\left|F\right|$ denotes the cardinality of feature set X and feature subset *F*. To guarantee satisfactory performance and overcome exhaustive experimentation, we require that AMI > $\hat{\mathrm{MI}}$ and ACMI > $\hat{\mathrm{CMI}}$ hold.

AKDB applies the greedy-search strategy to iteratively identify redundant and near-redundant features. For feature selection, we take advantage of the feature order that is determined by comparing MI. For simplicity, we adaptively provide the threshold value of MI that cuts off an entire region at the end of the order. Let δ be a user-specified parameter, 0%<δ≤100% (see detail in Section 4.1). We suppose that the difference between features $X_{i}$ and $X_{j}$ is non-significant if $I\left(X_{i};C\right)\leq I\left(X_{j};C\right)\leq I\left(X_{i};C\right)*\left(1+\delta \right)$. Correspondingly, we regard the feature $X_{j}$ as near-redundant if $X_{i}$ is redundant and the difference between features $X_{i}$ and $X_{j}$ is non-significant. Given the feature order, AKDB firstly selects the feature, e.g., $X_{i}$, at the end of the order and identifies it as a redundant feature. Then, we identify the near-redundant features. Finally, LOOCV is introduced to evaluate the classification performance after removing redundant and near-redundant features as it can provide the out-of-sample error with an unbiased low-variance estimation. In addition, the 0–1 loss is used as a loss function since it is an effective measure to evaluate the quality of a classifier’s classification performance.

Finally, the feature subset is selected with the lowest 0–1 loss. In case of a draw, preference is given to the smallest number of features. If the MI values are distributed densely, then all redundant and near-redundant features can be identified only in a few iterations. After that, the greedy-search strategy is applied to identify redundant and near-redundant conditional dependencies. In this paper, we proposed to extend KDB by using information-threshold based techniques, FeatureSelection (FS) and DependenceSelection (DS), to respectively identify redundant features and redundant conditional dependencies. Both techniques are based on backward elimination that begins at the full set of features or conditional dependencies.

The learning procedure of FS is shown in Algorithm 2. By applying BSE, FS aims to seeks a subset of the available features that minimizes 0–1 loss on the training set. FS starts from the full set of features and corresponding MI values have been grouped into several batches. There should exist significant differences between the MI values in different batches. Suppose that, for successive batches $B_{i}$ and $B_{i+1}$, $I_{m}=\min\left\{I\left(X_{j};C\right)\right\}$ for any $I\left(X_{j};C\right)\in B_{i}$ and $I_{m+1}=\min\left\{I\left(X_{k};C\right)\right\}$ for any $I\left(X_{k};C\right)\in B_{i+1}$. In this paper, FS requires that, for batches $B_{i}$ and $B_{i+1}$, the criterion $I_{m}*\left(1+\delta \right)<I_{m+1}$ holds, or for batch $B_{i}$ the criterion $I_{m}\leq I\left(X_{j};C\right)\leq I_{m}*\left(1+\delta \right)$ holds. BSE operates by iteratively removing successive batches. Then, the threshold of MI, or $\hat{\mathrm{MI}}$, will change from $I_{m}$ to $I_{m+1}$ if the removal can help reduce the 0–1 loss. The features in the batch or corresponding direct dependencies will be removed from the network structure and the classification performance will be evaluated iteratively using LOOCV. This procedure will terminate if there is no 0–1 loss improvement or $I_{m}$ > AMI.

When the learning procedure of FS terminates, DS is applied to identify non-significant conditional dependencies and its learning procedure is similar except that CMI rather than MI values will be grouped into several batches and we need to remove batch of CMI values iteratively to improve 0–1 loss. The learning procedure of DS is shown in Algorithm 3.

The description of a complete AKDB algorithm, which includes FS and DS techniques, is shown in Algorithm 4. Both FS and DS firstly apply the filter approach to rank feature or conditional dependence by MI or CMI criteria, then use the wrapper approach to evaluate the feature subset or dependence subset every time for better 0–1 loss results.

**Algorithm 2:** FeatureSelection(T, BN, L, AMI)

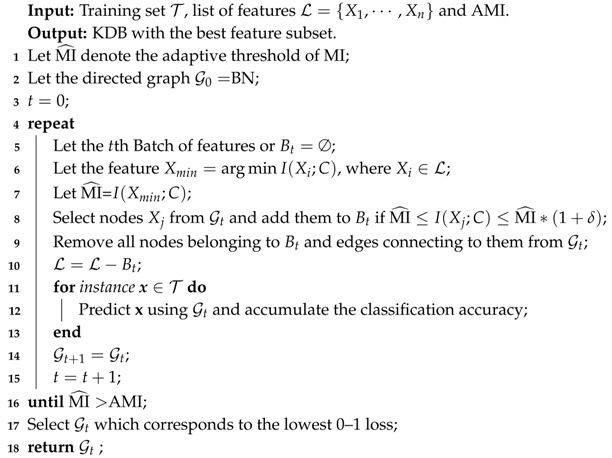



**Algorithm 3:** DependenceSelection(T, G, ACMI)

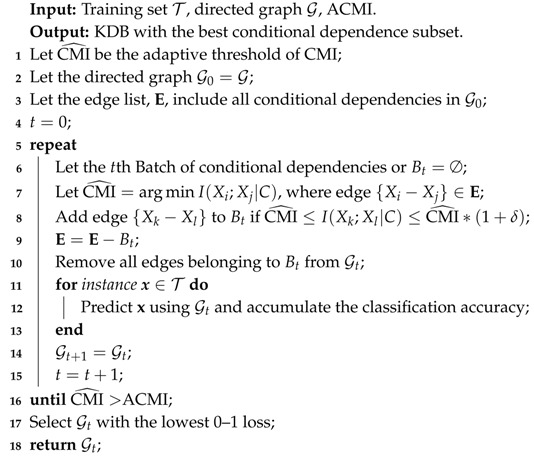



**Algorithm 4:** AKDB **Input**: Training set T with features $L=\{X_{1},\cdots ,X_{n},C\}$ and *k*. **Output**: AKDB model. **1** Calculate $I\left(X_{i};C\right)\left(1\leq i\leq n\right)$ from T for each feature and AMI; **2** Calculate $I\left(X_{i};X_{j}|C\right)\left(i\neq j\right)$ from T for every pair of features and ACMI; **3** Let L be a list which includes all $X_{i}$ in decreasing order of $I(X_{i};C)$; **4** Initialize the network structure G = LearnStructure(T,L,k);         // Algorithm 1 **5**
G = FeatureSelection(T,G,L, AMI);                      // Algorithm 2 **6**
G = DependenceSelection(T,G, ACMI);                    // Algorithm 3 **7 return**
G;

## 4. Experiments

We conduct the experiments on 30 benchmark datasets from UCI (University of California, Irvine) machine learning repository [34]. The detailed characteristics of these datasets are described in Table 2, which includes the number of instance, feature and class. The datasets are divided into two categories—first, small datasets with number of instances ≤3000; second, large datasets with number of instances >3000. Numeric features, if they exist in a dataset, are discretized based on Minimum Description Length (MDL) [35]. Missing values are considered as a distinct value and the *m*-estimation with m=1 [36] is employed to smooth the probability estimates.

The following algorithms are compared:

- NB, standard Naive Bayes.
- TAN, tree-augmented naive Bayes.
- NB-FSS, selective Naive Bayes classifier with forward sequential selection.
- K${}_{1}$DB, standard *k*-dependence Bayesian classifier with *k* = 1.
- K${}_{2}$DB, standard *k*-dependence Bayesian classifier with *k* = 2.
- AKDB, KDB with feature selection and conditional dependence selection based on adaptive thresholding.

The classification accuracy of algorithms are compared in terms of 0–1 loss and RMSE, and the results of them are respectively presented in Table A5 and Table A6. The bias and variance results are respectively provided in Table A7 and Table A8 because the bias-variance decomposition can provide valuable insights into the components of the error of learned algorithms [37,38]. Note that only 13 large datasets are selected because of statistical significance in terms of bias-variance comparison.

### 4.1. Selection of the Value of Parameter for AKDB

Removing redundant features or conditional dependencies from BNC may positively affect its classification performance if the threshold value δ is selected appropriately. However, there is no priori work that can achieve this goal. We perform an empirical study to select an appropriate δ. The 0–1 loss results for all datasets with different δ values are presented in Table 3. We can see that AKDB achieves the lowest 0–1 loss results more often when δ=10%. Although on some datasets AKDB with δ=10% may perform relatively poorer, the difference between the 0–1 loss when δ=10% and the lowest 0–1 loss is not significant (less than 5%). For example, on dataset Splice-C4.5, AKDB achieves the lowest 0–1 loss (0.0468) when δ=80%, and when δ=10% the 0–1 loss is 0.0469. From the experimental results, we argue that δ=10% is appropriate to help identify the threshold efficiently.

### 4.2. Effects of Feature Selection and Conditional Dependence Selection on KDB

FS and DS are two information-threshold based techniques which are used in the proposed algorithm AKDB. Using these techniques will cause a portion of features and conditional dependencies to be removed. To prove that they can work severally, we present respectively two versions of KDB as follows:

- KDB-FS, KDB with only feature selection,
- KDB-DS, KDB with only conditional dependence selection.

In order to evaluate the difference between two classifiers, we define the relative ratio as follows:

(5) $$R_{M}\left(A|B\right)=1-\frac{M_{A}}{M_{B}}.$$

The values of parameter *M* represents different measures. Corresponding values of $R_{M}\left(A|B\right)$ represent the difference in percentage between two classifiers *A* and *B* based on parameter *M*.

**Figure 5:**
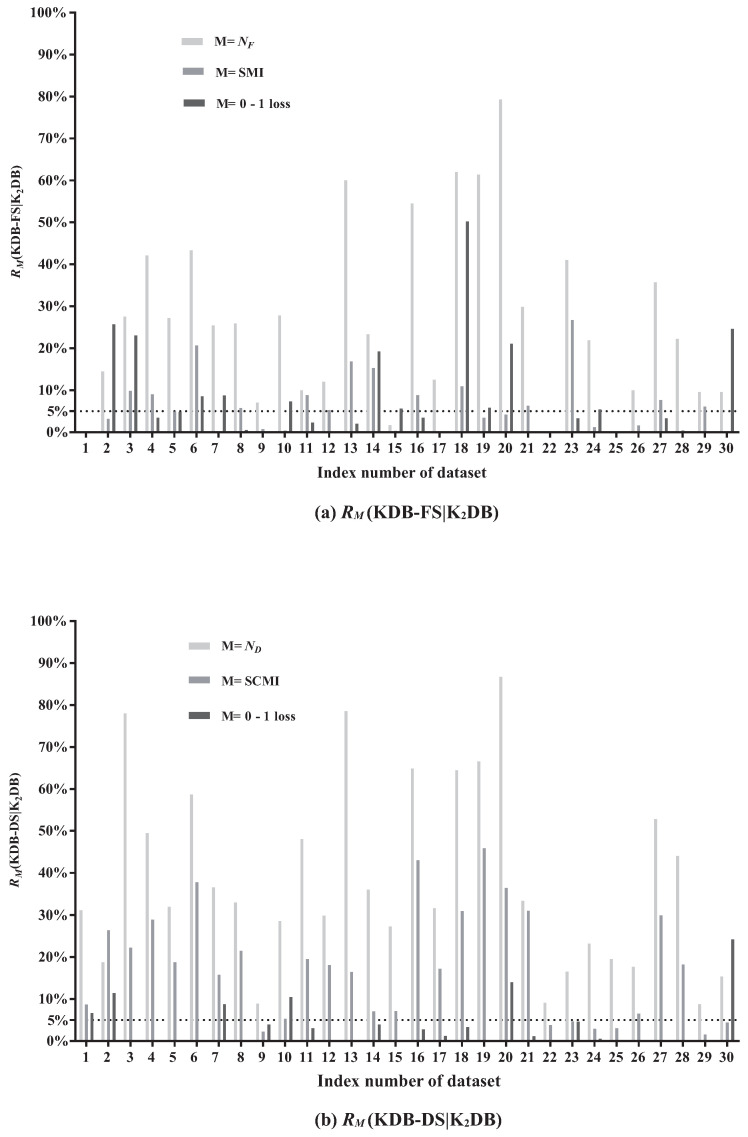
The comparison results of $R_{M}$(KDB-FS|K${}_{2}$DB) and $R_{M}$(KDB-DS|K${}_{2}$DB).

In this paper, $N_{F}$ and $N_{D}$ are respectively used to denote the number of features and the number of conditional dependencies in BNC. SMI and SCMI are used to indicate the sum of MI and CMI in BNC, respectively. The results of $R_{M}$(KDB-FS|K${}_{2}$DB) and $R_{M}$(KDB-DS|K${}_{2}$DB) are shown in Figure 5a,b, respectively.

Figure 5a presents relative ratios between KDB-FS and K${}_{2}$DB in terms of $N_{F}$, SMI and 0–1 loss. The effectiveness of FS can be demonstrated by comparing the SMI values before and after removing redundant features. From Figure 5a, FS removes features on 27 out of 30 datasets. The larger the value of $R_{N_{F}}$(KDB-FS|K${}_{2}$DB), the more features that are identified as redundant and removed. We can see that the values of $R_{N_{F}}$(KDB-FS|K${}_{2}$DB) on five datasets are greater than 50%. For example, on dataset Hypo (No. 20), $R_{N_{F}}$(KDB-FS|K${}_{2}$DB) = 79.31%, indicating that 79.31% of features are identified as redundant and removed. The AMI value on dataset Hypo with 29 features is 0.0251 and only three features have MI values greater than the AMI value. In addition, 23 of these 29 features have MI values lower than 0.007 and they are iteratively removed from KDB according to the greedy-search strategy. Thus, the significant difference in MI values contributes to this high value of $R_{N_{F}}$(KDB-FS|K${}_{2}$DB). Furthermore, removing features based on the FS technique will not result in strong direct dependencies to be removed. For example, the value of $R_{\mathrm{SMI}}$(KDB-FS|K${}_{2}$DB) on datasets Hypo is 4.14%, although 79.31% of features are removed. On dataset Wavement (No. 30), the value of $R_{\mathrm{SMI}}$(KDB-FS|K${}_{2}$DB) is close to 0% after removing 9.52% of features. These facts suggest that those removed features in KDB show weak direct dependencies. In addition, removing weak direct dependencies may help improve the classification performance. The values of $R_{0\text{-}1\;\mathrm{Loss}}$(KDB-FS|K${}_{2}$DB) on datasets Hypo and Wavement are 21.05% and 24.61%, respectively. That is, the classification performance is improved after removing the weak direct dependencies. The significant improvement in 0–1 loss (the value of $R_{0\text{-}1\;\mathrm{Loss}}$(•) > 5%) on 12 datasets has demonstrated that the FS technique demonstrates a positive influence on classification performance.

The redundancy of conditional dependencies may also exist in KDB. Figure 5b presents the relative ratios between KDB-DS and K${}_{2}$DB in terms of $N_{D}$, SCMI and 0–1 loss. The comparison of the SCMI values before and after removing redundant conditional dependencies can demonstrate the effectiveness of DS. When DS is applied to KDB, the selection of conditional dependencies occurs on all 30 datasets. The value of $R_{N_{D}}$(KDB-DS|K${}_{2}$DB) ranges from 8.77% to 86.72%. The larger the value of $R_{N_{D}}$(KDB-DS|K${}_{2}$DB), the more conditional dependencies that are identified as redundant and removed. For example, the value of $R_{N_{D}}$(KDB-DS|K${}_{2}$DB) is 78.51% on dataset Credit-a. It indicates that on average only 5.8 of 27 conditional dependencies are retained. Furthermore, 24 of all 27 CMI values are lower than the ACMI value (0.1592), and even the minimum CMI value is 0.0189. The difference in CMI values on dataset Credit-a is obvious; by applying the DS technique with the greedy-search strategy, weak conditional dependencies are iteratively removed. The value of $R_{\mathrm{SCMI}}$(KDB-DS|K${}_{2}$DB) ranges from 1.54% to 45.83%. The high value of $R_{\mathrm{SCMI}}$(KDB-DS|K${}_{2}$DB) does not indicate that the strong conditional dependencies are removed. On dataset Hypo, $R_{\mathrm{SCMI}}$(KDB-DS|K${}_{2}$DB) = 36.40%. The factor that contributes to this high value is that the SCMI value of these 48 removed conditional dependencies reaches 2.3355, but the CMI value of each removed conditional dependence is lower than the ACMI value. When it comes to 0–1 loss, the value of $R_{0\text{-}1\;\mathrm{Loss}}$(KDB-DS|K${}_{2}$DB) on dataset Hypo is 14.04%. It indicates that deleting those weak conditional dependencies may help improve classification accuracy. KDB-DS achieves almost the same classification accuracy as K${}_{2}$DB with a simplified network structure on 14 datasets and achieves 0–1 loss improvement on 16 datasets. These results indicate that the DS technique is effective and can help reduce the structure complexity of KDB.

Both FS and DS techniques combine the characteristics of filter and wrapper approaches. The redundant features or conditional dependencies are filtered out and then we use classification accuracy to evaluate the feature subsets or the retained conditional dependencies, respectively. On the other hand, removing redundant features and conditional dependencies can reduce the parameters that are needed for probability estimates and may improve the classification accuracy. From the above discussion, we can see that both FS and DS techniques are efficient and can help improve the classification performance.

### 4.3. Comparison of AKDB vs. NB, NB-FSS, TAN, K${}_{1}$DB and K${}_{2}$DB

In this section, we conduct comparisons for related algorithms in terms of 0–1 loss, RMSE and bias-variance decomposition. RMSE [2] is computed as:

(6) $$RMSE=\sqrt{\frac{1}{t}\sum_{x\epsilon T}{\left(1-\hat{p}\left(c^{x}|x\right)\right)}^{2}},$$

where *t* is the number of training instances in training set T, $c^{x}$ is the true class label for the instance x, and $\hat{p}\left(c^{x}|x\right)$ is the estimated posterior probability of the true class given x.

The win/draw/loss (W/D/L) records of 0–1 loss, RMSE and bias-variance decomposition are presented in Table 4, Table 5 and Table 6, respectively. The W/D/L record of the comparison results of every two different algorithms are presented in each cell [i;j] of every table. When one algorithm in row *i* ($Al_{i}$) and the another algorithm in column *j* ($Al_{j}$) are compared, we can observe which algorithm performs better on all datasets from cell [i;j]. This is because, in cell [i;j], a win denotes that $Al_{i}$ obtains a lower 0–1 loss than $Al_{j}$, a loss denotes $Al_{j}$ that obtains a lower 0–1 loss than $Al_{i}$, and a draw denotes that $Al_{i}$ and $Al_{j}$ perform comparably. We regard a difference as significant if the outcome of a one-tailed binomial sign test is less than 0.05 [39,40].

From Table 4, we can see that NB-FSS performs better than NB in terms of a 0–1 loss. It indicates that FSS is feasible to NB. Surprisingly, K${}_{2}$DB does not have an obvious advantage when compared to 1-dependence classifiers. In addition, it even performs poorer when compared to TAN. However, when it comes to large datasets, as Table 7 shows, K${}_{2}$DB performs better than both TAN and K${}_{1}$DB. We can see that AKDB significantly outperforms all other algorithms. Most importantly, when compared to K${}_{2}$DB, AKDB has a 0–1 loss improvement with 15 wins and only one loss, which proves that the proposed two information-threshold based techniques are effective. This advantage is even greater on small datasets. From Table A5, AKDB never loses on small datasets and it obtains a significantly lower 0–1 loss on 11 out of 17 small datasets. On dataset Lymphography, the error is substantially reduced from 0.2365 to 0.1554. Compared to K${}_{2}$DB on large datasets, AKDB achieves W/D/L record of 4/8/1. Although the improvement is not significant, AKDB only loses on dataset Spambase. Based on these facts, we argue that AKDB is a more effective algorithm in terms of 0–1 loss.

What is revealed in Table 5 is similar to that in Table 4. NB and NB-FSS perform worse, which demonstrates the limitations of the independence assumption in NB. TAN and K${}_{1}$DB get better performance than NB and NB-FSS. In addition, AKDB still achieves lower RMSE significantly more often than the other five algorithms. On average, 72.4% of the features and 59.6% of conditional dependencies are selected to build the network structure of AKDB, although in some cases the improvement in terms of RMSE is not significant. Considering that AKDB has significantly lower 0–1 loss and RMSE in comparison to other algorithms, we argue that the FS technique in tandem with the DS technique used in the proposed algorithm is powerful to improve classification accuracy.

The W/D/L records of bias-variance decomposition are presented in Table 6. We may observe that NB and NB-FSS achieve higher bias and lower variance significantly more often than the other algorithms because their structures are definite without considering the true data distribution. TAN, K${}_{1}$DB and K${}_{2}$DB are all low-bias and high-variance learners because they are derived from higher-dimensional probability estimates. Thus, these classifiers are more sensitive to the changes in the training data. AKDB performs best in terms of bias. When compared to K${}_{1}$DB and K${}_{2}$DB, AKDB obtains lower bias more often than them, as jointly applying both FS and DS to KDB can simplify the network structure. Furthermore, we can observe that AKDB shows an advantage over K${}_{2}$DB in variance. The average of variance of K${}_{2}$DB and AKDB are 0.045 and 0.025 on 13 large datasets, respectively. Based on these facts, we argue that the proposed AKDB is more stable for classification.

### 4.4. Tests of Significant Differences

Friedman proposed the Friedman test [41] for comparisons of multiple algorithms over multiple datasets. It first calculates the ranks of algorithms for each dataset separately, and then compares the average ranks of algorithms over datasets. The best performance algorithm getting the rank of 1, the second best rank of 2, and so on. The null-hypothesis is that there is no significant difference in terms of average ranks. The Friedman test is a non-parametric measure which can be computed as follows:

(7) $$\chi_{F}^{2}=\frac{12}{Nt(t+1)}\sum_{j=1}^{t}R_{j}^{2}-3N\left(t+1\right),$$

where *N* and *t* respectively denote the number of datasets and the number of algorithms, and $R_{j}$ is the average rank of the *j*-th algorithm. With the 30 datasets and 6 (*t* = 6) algorithms, the critical value of $\chi_{\alpha }^{2}$ for α=0.05 with (*t*− 1) degrees of freedom is 11.07. The Friedman statistics $\chi_{F}^{2}$ of experimental results in Table A5 and Table A6 are respectively 36.56 and 22.90, which are larger than $\chi_{\alpha }^{2}$, 11.07. Hence, we reject all the null-hypothesis.

**Figure 6:**
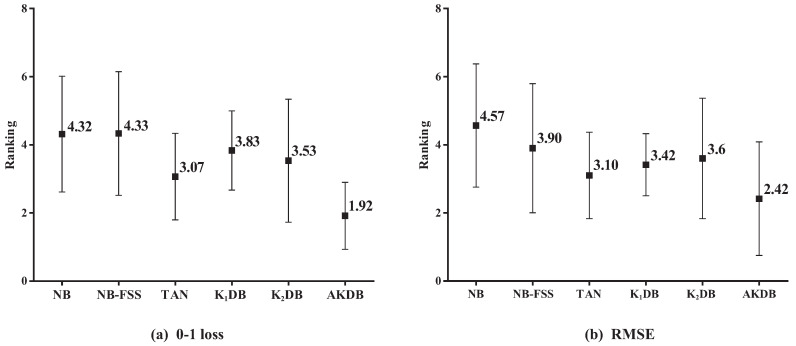
The results of ranking in terms of 0–1 loss and RMSE for alternative algorithms.

Figure 6 presents the results of average ranking in terms of 0–1 loss and RMSE for six algorithms. The average ranks of different algorithms based on 0–1 loss on all datasets are, respectively, {NB(4.32), NB-FSS(4.33), K${}_{1}$DB(3.07), TAN(3.83), K${}_{2}$DB(3.53), and AKDB(1.92)}. That is, the ranking of AKDB is higher than that of other algorithms, followed by TAN, K${}_{2}$DB, K${}_{1}$DB, NB, and NB-FSS. When assessing performance using RMSE, AKDB still obtains the advantage of ranking with the lowest average rank, i.e., 2.42.

**Figure 7:**
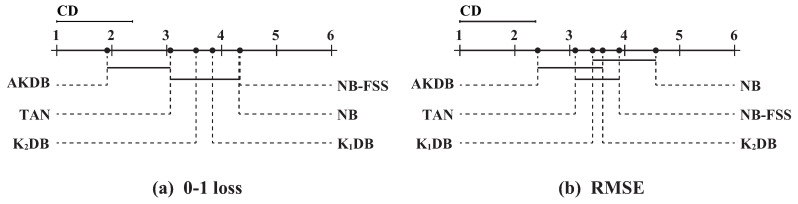
The results of Nemenyi tests in terms of 0–1 loss and RMSE for alternative algorithms.

In order to determine which algorithm has a significant difference to others, we further employ the Nemenyi test [42]. The comparisons of six algorithms against each other with the Nemenyi test on 0–1 loss and RMSE are shown in Figure 7. Critical difference (CD) is also presented in the figure that is calculated as follows:

(8) $$CD=q_{\alpha }\sqrt{\frac{t(t+1)}{6N}},$$

where the critical value $q_{\alpha }$ for α=0.05 and t=6 is 2.85. With the 30 (*N* = 30) datasets and six algorithms, CD = $2.85\times \sqrt{6\times (6+1)/(6\times 30)}=$ 1.377. On the top dotted line, we plot the algorithms based on their average ranks, which are indicated on the top solid line. On a line, the lower rank is to the more leftward position and the algorithm on the left side has better performance. The algorithms are connected by a line if their differences are not significant.

As shown in Figure 7a, these algorithms are divided into two groups clearly in terms of 0–1 loss. One group includes AKDB and TAN, and other algorithms are in another group. AKDB ranks first although it does not have a significant advantage when compared to TAN. AKDB enjoys a significant 0–1 loss advantage relative to K${}_{2}$DB, K${}_{1}$DB, NB and NB-FSS, proving the effectiveness of the proposed information-threshold based techniques in our algorithm. As shown in Figure 7b, when RMSE is compared, AKDB still achieves lower mean ranks than the other algorithms, although the differences between AKDB, K${}_{1}$DB, K${}_{2}$DB are not significant.

## 5. Discussion

KDB is a form of restricted BNCs, and the weak direct dependencies and conditional dependencies may exist in KDB and they may be redundant. To alleviate the potential redundancy problem, we develop an extension to KDB, called AKDB, which applies feature selection and conditional dependence selection to remove redundant features and conditional dependencies. These two techniques presented in this paper, MI-based feature selection and CMI-based dependence selection, are based on adaptive thresholding. They are designed to iteratively identify relevant features and conditional dependencies in certain circumstances, and they combine the characteristics of filter and wrapper approaches. Both techniques are efficient and complementary. By providing experiments on 30 UCI datasets and comparisons with other state-of-the-art BNCs, we prove that adaptive thresholding can help select the most relevant features and conditional dependencies with an improvement in classification performance. On average, 72.4% of the features and 59.6% of conditional dependencies are selected to build the network structure of AKDB. Overall, AKDB achieves significant advantage over KDB in terms of 0–1 loss by a 8.54% reduction on average. The statistical significance of the experiment results is further confirmed by the Friedman test and Nemenyi test.

## 6. Conclusions

To efficiently identify non-significant direct and conditional dependencies, we investigate two techniques to extend KDB, MI-based feature selection and CMI-based dependence selection based on adaptive thresholding. These two techniques combine the characteristics of filter and wrapper approaches and when applied to KDB, they are severally efficient for filtering out redundancy and can help improve the classification performance. The extensive experimental results show that the final classifier, AKDB, significantly outperforms several state-of-the-art BNCs, including NB, TAN and KDB.

## Figures and Tables

**Table 1:** List of acronyms used.

Notation	Description
MI	mutual information
CMI	conditional mutual information
BNCs	Bayesian network classifiers
BNC	Bayesian network classifier
*B*	a BNC
LOOCV	leave-one-out cross validation
RMSE	root mean squared error
AMI	the average MI
ACMI	the average CMI
FS	feature selection
DS	dependence selection
MDL	Minimum Description Length
$N_{F}$	the number of features in BNC
$N_{D}$	the number of conditional dependencies in BNC
SMI	the sum of MI
SCMI	the sum of CMI

**Table 2:** Description of the datasets used in the experiments.

No.	Dataset	Instance	Feature	Class	No.	Dataset	Instance	Feature	Class
1	Echocardiogram	131	6	2	16	German	1000	20	2
2	Lymphography	148	18	4	17	Yeast	1484	8	10
3	Iris	150	4	3	18	Splice-c4.5	3177	60	3
4	Hepatitis	155	19	2	19	Dis	3772	29	2
5	Autos	205	25	7	20	Hypo	3772	29	4
6	Glass Identification	214	9	3	21	Spambase	4601	57	2
7	Heart	270	13	2	22	Phoneme	5438	7	50
8	Primary Tumor	339	17	22	23	Page-blocks	5473	10	5
9	Ionosphere	351	34	2	24	Optdigits	5620	64	10
10	Musk1	476	166	2	25	Mushroom	8124	22	2
11	Balance-scale	625	4	3	26	Magic	19,020	10	2
12	Soybean	683	35	19	27	Adult	48,842	14	2
13	Credit-a	690	15	2	28	Shuttle	58,000	9	7
14	Breast-cancer-w	699	9	2	29	Connect-4	67,557	42	3
15	Vehicle	846	18	4	30	Waveform	100,000	21	3

**Table 3:** The 0–1 loss results of AKDB for all datasets with different δ values.

Dataset	δ=5%	δ=10%	δ=20%	δ=30%	δ=40%	δ=50%	δ=60%	δ=70%	δ=80%	δ=90%
Echocardiogram	0.3740	**0.3206**	0.3359	0.3511	0.3588	0.3664	0.3740	0.3664	0.3664	0.3664
Lymphography	0.2365	**0.1554**	0.2432	0.2095	0.2162	0.2027	0.2568	0.2027	0.2162	0.2500
Iris	0.0867	**0.0733**	0.0867	0.0867	0.0800	0.0767	0.0767	0.0800	**0.0733**	**0.0733**
Hepatitis	0.1677	**0.1419**	0.2129	0.1935	0.2323	0.2129	0.2000	0.2323	0.2323	0.2323
Autos	0.2098	**0.1951**	0.2000	0.2098	0.2098	0.1951	0.2195	0.2000	0.2000	0.2000
Glass-Id	0.2103	**0.1963**	0.2103	0.2150	0.2196	0.2243	0.2150	0.2196	0.2103	0.2150
Heart	0.1963	**0.1630**	0.1963	0.1963	0.1963	0.1889	0.1852	0.1852	0.1815	0.1815
Primary-Tumor	0.5693	**0.5428**	0.5988	0.5988	0.5693	0.5664	0.5811	0.5841	0.5752	0.5782
Ionosphere	0.0912	**0.0712**	0.0883	0.0912	0.0855	0.0769	0.0912	0.0940	0.0826	0.0940
Musk1	0.1176	**0.1029**	0.1134	0.1155	0.1155	0.1092	0.1197	0.1197	0.1218	0.1261
Balance-Scale	0.2784	0.2800	0.2784	0.2784	0.2784	0.2784	0.2784	0.2784	0.2784	**0.2752**
Soybean	0.0556	**0.0527**	0.0556	0.0600	0.0571	0.0630	0.0571	0.0732	0.0761	0.1098
Credit-A	0.1681	**0.1420**	0.1551	0.1609	0.1768	0.1638	0.1493	0.1623	0.1536	0.1522
Breast-Cancer-W	0.0744	**0.0472**	0.0544	0.0644	0.0730	0.0758	0.0758	0.0758	0.0758	0.0601
Vehicle	**0.2983**	0.3014	0.2996	0.2986	0.2990	0.3002	0.3168	0.3109	0.3322	0.3310
German	0.2920	**0.2590**	0.2700	0.2920	0.2880	0.2890	0.2880	0.2940	0.2950	0.2940
Yeast	0.4387	**0.4218**	0.4447	0.4468	0.4461	0.4461	0.4501	0.4569	0.4616	0.4778
Splice-C4.5	0.0853	0.0469	0.0661	0.0585	0.0516	0.0529	0.0475	0.0475	**0.0468**	**0.0468**
Dis	0.0151	**0.0130**	0.0146	0.0154	0.0151	0.0146	0.0138	0.0143	0.0146	0.0151
Hypo	0.0130	**0.0077**	0.0130	0.0103	0.0170	0.0217	0.0225	0.0233	0.0225	0.0233
Spambase	0.0762	**0.0752**	0.0767	0.0796	0.0761	0.0776	0.0785	0.0795	0.0787	0.0813
Phoneme	**0.1984**	**0.1984**	**0.1984**	**0.1984**	0.2896	0.2602	0.2655	0.2519	0.2589	0.2758
Page-Blocks	0.0391	0.0391	0.0391	0.0391	0.0391	**0.0376**	0.0380	0.0389	0.0402	0.0386
Optdigits	0.0438	**0.0358**	0.0372	0.0368	0.0391	0.0388	0.0374	0.0368	0.0400	0.0418
Mushrooms	0.0004	**0.0000**	**0.0000**	**0.0000**	**0.0000**	0.0004	**0.0000**	**0.0000**	0.0010	0.0011
Magic	0.1637	**0.1636**	0.1722	0.1900	0.1904	0.1914	0.1909	0.1906	0.1917	0.1897
Adult	0.1375	0.1338	0.1338	**0.1337**	0.1347	0.1347	0.1348	0.1409	0.1421	0.1413
Shuttle	**0.0009**	**0.0009**	0.0018	0.0018	0.0018	0.0021	0.0018	0.0018	0.0018	0.0019
Connect-4	0.2294	**0.2283**	0.2442	0.2499	0.2535	0.2591	0.2575	0.2592	0.2652	0.2692
Waveform	**0.0193**	0.0196	**0.0193**	0.0195	0.0194	0.0194	0.0194	0.0194	0.0194	0.0234

The lowest 0–1 loss results for datasets are shown in bold.

**Table 4:** W/D/L records of 0–1 loss on all datasets.

W/D/L	NB	NB-FSS	TAN	K${}_{1}$DB	K${}_{2}$DB
NB-FSS	10/12/8				
TAN	17/8/5	18/6/6			
K${}_{1}$DB	19/5/6	19/5/6	5/18/7		
K${}_{2}$DB	17/7/6	20/1/9	8/11/11	11/11/8	
AKDB	21/7/2	22/5/3	16/11/3	19/11/0	15/14/1

**Table 5:** W/D/L records of RMSE on all datasets.

W/D/L	NB	NB-FSS	TAN	K${}_{1}$DB	K${}_{2}$DB
NB-FSS	6/18/6				
TAN	17/10/3	14/9/7			
K${}_{1}$DB	18/10/2	15/10/5	2/25/3		
K${}_{2}$DB	16/9/5	16/7/7	6/19/5	6/18/6	
AKDB	20/8/2	18/6/6	10/13/7	8/18/4	7/21/2

**Table 6:** W/D/L records of bias and variance on large datasets.

	W/D/L	NB	NB-FSS	TAN	K${}_{1}$DB	K${}_{2}$DB
	NB-FSS	6/3/4				
	TAN	9/1/3	10/1/2			
Bias	K${}_{1}$DB	11/1/1	12/1/0	4/5/4		
	K${}_{2}$DB	11/0/2	12/0/1	6/4/3	7/3/3	
	AKDB	11/1/1	11/2/0	8/2/3	8/1/4	5/3/5
	NB-FSS	13/0/0				
	TAN	4/0/9	0/0/13			
Variance	K${}_{1}$DB	4/2/7	0/0/12	4/3/6		
	K${}_{2}$DB	5/0/8	0/0/13	4/1/8	4/0/9	
	AKDB	8/0/5	1/1/11	9/1/3	10/2/1	12/0/1

**Table 7:** W/D/L records of 0–1 loss on large datasets.

W/D/L	NB	NB-FSS	TAN	K${}_{1}$DB	K${}_{2}$DB
NB-FSS	5/5/3				
TAN	9/4/0	10/1/2			
K${}_{1}$DB	11/1/1	10/2/1	3/7/3		
K${}_{2}$DB	11/0/2	12/0/1	8/3/2	9/1/3	
AKDB	12/0/1	11/1/1	7/5/1	9/4/0	4/8/1

## Appendix A

**Table A1:** The values of $I(X_{i};C)$ for each feature in K${}_{2}$DB on Hypo dataset.

No.	MI	No.	MI	No.	MI	No.	MI	No.	MI
1	0.0000	7	0.0002	13	0.0009	19	0.0020	25	0.0123
2	0.0000	8	0.0004	14	0.0012	20	0.0030	26	0.0337
3	0.0000	9	0.0005	15	0.0017	21	0.0052	27	0.1425
4	0.0000	10	0.0006	16	0.0017	22	0.0062	28	0.1580
5	0.0001	11	0.0007	17	0.0018	23	0.0065	29	0.3528
6	0.0001	12	0.0007	18	0.0019	24	0.0105		

**Table A2:** The values of $I(X_{i};C)$ for each feature in K${}_{2}$DB on Waveform dataset.

No.	MI	No.	MI	No.	MI	No.	MI	No.	MI
1	0.0000	6	0.5847	11	0.6348	16	0.6588	21	0.7497
2	0.0000	7	0.6014	12	0.6379	17	0.7023		
3	0.4891	8	0.6020	13	0.6439	18	0.7111		
4	0.4960	9	0.6295	14	0.6446	19	0.7400		
5	0.5801	10	0.6305	15	0.6550	20	0.7424		

**Table A3:** The results of $I(X_{i};X_{j}|C)$ for each feature pair in K${}_{2}$DB on Hypo dataset.

No.	CMI	No.	CMI	No.	CMI	No.	CMI	No.	CMI
1	0.0000	12	0.0035	23	0.0092	34	0.0355	45	0.2331
2	0.0000	13	0.0044	24	0.0092	35	0.0471	46	0.3031
3	0.0000	14	0.0048	25	0.0098	36	0.0864	47	0.3354
4	0.0000	15	0.0049	26	0.0121	37	0.1153	48	0.4571
5	0.0000	16	0.0058	27	0.0146	38	0.1189	49	0.4782
6	0.0000	17	0.0058	28	0.0154	39	0.1240	50	0.4786
7	0.0018	18	0.0064	29	0.0241	40	0.1313	51	0.4834
8	0.0019	19	0.0073	30	0.0262	41	0.1361	52	0.4852
9	0.0023	20	0.0073	31	0.0279	42	0.1390	53	0.4912
10	0.0024	21	0.0076	32	0.0279	43	0.1855	54	0.5099
11	0.0032	22	0.0090	33	0.0286	44	0.2007	55	0.7263

**Table A4:** The results of $I(X_{i};X_{j}|C)$ for each feature pair in K${}_{2}$DB on Waveform dataset.

No.	CMI	No.	CMI	No.	CMI	No.	CMI	No.	CMI
1	0.0000	9	0.0029	17	0.1024	25	0.2741	33	0.4917
2	0.0000	10	0.0530	18	0.1408	26	0.3046	34	0.5233
3	0.0000	11	0.0580	19	0.1463	27	0.3077	35	0.5291
4	0.0000	12	0.0750	20	0.1510	28	0.3922	36	0.5318
5	0.0016	13	0.0872	21	0.1548	29	0.4092	37	0.5449
6	0.0016	14	0.0937	22	0.1611	30	0.4115	38	0.5748
7	0.0017	15	0.0969	23	0.1612	31	0.4564	39	0.5847
8	0.0022	16	0.0973	24	0.2475	32	0.4752		

**Table A5:** Experimental results of 0–1 loss.

Dataset	NB	SNB-FSS	TAN	K${}_{1}$DB	K${}_{2}$DB	KDB-FS	KDB-DS	AKDB
Echocardiogram	0.3359	0.3664	0.3282	0.3053	0.3435	0.3435	0.3206	0.3206 ∘
Lymphography	0.1486	0.1689	0.1757	0.1757	0.2365	0.1757	0.2095	0.1554 ∘
Iris	0.0867	0.0600	0.0800	0.0867	0.0867	0.0667	0.0867	0.0733 ∘
Hepatitis	0.1935	0.1677	0.1677	0.1548	0.1871	0.1806	0.1871	0.1419 ∘
Autos	0.3122	0.3561	0.2146	0.2146	0.2049	0.1951	0.2049	0.1951
Glass-id	0.2617	0.2430	0.2196	0.2243	0.2196	0.2009	0.2196	0.1963 ∘
Heart	0.1778	0.1741	0.1926	0.1963	0.2111	0.1926	0.1926	0.1630 ∘
Primary-tumor	0.5457	0.5398	0.5428	0.5693	0.5723	0.5693	0.5723	0.5428 ∘
Ionosphere	0.1054	0.0826	0.0684	0.0741	0.0741	0.0741	0.0712	0.0712
Musk1	0.1660	0.1450	0.1134	0.1113	0.1155	0.1071	0.1034	0.1029 ∘
Balance-scale	0.2720	0.3648	0.2736	0.2816	0.2784	0.2720	0.2700	0.2800
Soybean	0.0893	0.0952	0.0469	0.0644	0.0556	0.0556	0.0556	0.0527 ∘
Credit-a	0.1406	0.1377	0.1507	0.1551	0.1464	0.1435	0.1464	0.1420
Breast-cancer-w	0.0258	0.0258	0.0415	0.0486	0.0744	0.0601	0.0715	0.0472 ∘
Vehicle	0.3924	0.4054	0.2943	0.3014	0.2943	0.2778	0.2943	0.3014
German	0.2530	0.2660	0.2730	0.2760	0.2890	0.2790	0.2810	0.2590 ∘
Yeast	0.4239	0.4239	0.4171	0.4218	0.4387	0.4387	0.4333	0.4218
Splice-c4.5	0.0444	0.0381	0.0466	0.0482	0.0941	0.0469	0.0910	0.0469 ∘
Dis	0.0159	0.0154	0.0159	0.0146	0.0138	0.0130	0.0138	0.0130 ∘
Hypo	0.0138	0.0244	0.0141	0.0077	0.0114	0.0090	0.0098	0.0077 ∘
Spambase	0.1015	0.0765	0.0669	0.0765	0.0635	0.0635	0.0628	0.0752 •
Phoneme	0.2615	0.2477	0.2733	0.2120	0.1984	0.1984	0.1984	0.1984
Page-blocks	0.0619	0.0442	0.0415	0.0433	0.0391	0.0378	0.0373	0.0391
Optdigits	0.0767	0.0788	0.0407	0.0416	0.0372	0.0352	0.0370	0.0358
Mushrooms	0.0196	0.0148	0.0001	0.0006	0.0000	0.0000	0.0000	0.0000
Magic	0.2239	0.2132	0.1675	0.1742	0.1637	0.1637	0.1636	0.1636
Adult	0.1592	0.1656	0.1380	0.1385	0.1383	0.1338	0.1383	0.1338
Shuttle	0.0039	0.0040	0.0015	0.0015	0.0009	0.0009	0.0009	0.0009
Connect-4	0.2783	0.2999	0.2354	0.2406	0.2283	0.2282	0.2283	0.2283
Waveform	0.0220	0.0273	0.0202	0.0226	0.0256	0.0193	0.0194	0.0196 ∘

∘, • denote significant improvement or degradation of AKDB over K${}_{2}$DB.

**Table A6:** Experimental results of RMSE.

Dataset	NB	SNB-FSS	TAN	K${}_{1}$DB	K${}_{2}$DB	AKDB
Echocardiogram	0.4896	0.4823	0.4886	0.4846	0.4889	0.4807
Lymphography	0.3465	0.3505	0.3813	0.3726	0.4362	0.4076
Iris	0.2545	0.2158	0.2441	0.2435	0.2447	0.2224
Hepatitis	0.3901	0.3770	0.3610	0.3559	0.3875	0.3823
Autos	0.5190	0.5330	0.4475	0.4460	0.4399	0.4380
Glass-id	0.4353	0.4325	0.4109	0.4223	0.4205	0.4105
Heart	0.3651	0.3579	0.3771	0.3752	0.3949	0.3773
Primary-tumor	0.7084	0.7159	0.7170	0.7190	0.7262	0.7092
Ionosphere	0.0856	0.0538	0.2615	0.0621	0.0499	0.0561
Musk1	0.3972	0.3839	0.3022	0.3034	0.3058	0.3034
Balance-scale	0.4431	0.5448	0.4344	0.4384	0.4323	0.4605
Soybean	0.2945	0.3845	0.2014	0.2206	0.2063	0.2223
Credit-a	0.3342	0.3179	0.3411	0.3400	0.3525	0.3391
Breast-cancer-w	0.1570	0.1570	0.1928	0.1951	0.2497	0.2199
Vehicle	0.5736	0.5663	0.4593	0.4623	0.4591	0.4419
German	0.4945	0.4212	0.5000	0.4991	0.5053	0.4644
Yeast	0.5987	0.5987	0.5994	0.5997	0.6035	0.6035
Splice-c4.5	0.1883	0.2030	0.1917	0.1944	0.2756	0.1848
Dis	0.1177	0.1104	0.1103	0.1072	0.1024	0.1024
Hypo	0.1105	0.1401	0.1050	0.0881	0.0955	0.0863
Spambase	0.2994	0.3939	0.2403	0.2480	0.2300	0.2300
Phoneme	0.4792	0.4632	0.5048	0.4385	0.4195	0.4195
Page-blocks	0.2331	0.1923	0.1894	0.1940	0.1811	0.1781
Optdigits	0.2637	0.2893	0.1906	0.1937	0.1806	0.1736
Mushrooms	0.1229	0.1083	0.0083	0.0188	0.0001	0.0001
Magic	0.3974	0.3802	0.3461	0.3509	0.3470	0.3470
Adult	0.3409	0.3345	0.3076	0.3071	0.3089	0.3047
Shuttle	0.0561	0.0674	0.0356	0.0367	0.0290	0.0279
Connect-4	0.4787	0.5024	0.4435	0.4480	0.4336	0.4206
Waveform	0.1441	0.1499	0.1164	0.1285	0.1402	0.1253

**Table A7:** Experimental results of Bias.

Dataset	NB	SNB-FSS	TAN	K${}_{1}$DB	K${}_{2}$DB	AKDB
Splice-c4.5	0.0341	0.0355	0.0444	0.0358	0.0968	0.0353
Dis	0.0160	0.0191	0.0188	0.0174	0.0171	0.0190
Hypo	0.0098	0.0177	0.0101	0.0083	0.0072	0.0077
Spambase	0.0965	0.0735	0.0656	0.0665	0.0504	0.0589
Phoneme	0.2284	0.2004	0.2470	0.1740	0.1599	0.1572
Page-blocks	0.0409	0.0363	0.0331	0.0342	0.028	0.0286
Optdigits	0.0655	0.0685	0.0308	0.0313	0.0285	0.0235
Mushrooms	0.0399	0.0148	0.0002	0.0011	0.0002	0.0000
Magic	0.1987	0.1942	0.1357	0.1451	0.1321	0.1292
Adult	0.1485	0.1880	0.1125	0.1117	0.1135	0.1236
Shuttle	0.0066	0.0036	0.0023	0.0026	0.0028	0.0008
Connect-4	0.2327	0.2959	0.1829	0.1882	0.1788	0.2069
Waveform	0.0314	0.0257	0.0138	0.0154	0.0180	0.0164

**Table A8:** Experimental results of Variance.

Dataset	NB	SNB-FSS	TAN	K${}_{1}$DB	K${}_{2}$DB	AKDB
Splice-c4.5	0.0095	0.0051	0.0296	0.0357	0.0813	0.0572
Dis	0.0091	0.0000	0.0009	0.0021	0.0025	0.0012
Hypo	0.0063	0.0033	0.0078	0.0066	0.0059	0.0069
Spambase	0.0104	0.0070	0.0171	0.0176	0.0238	0.0178
Phoneme	0.1831	0.0783	0.2496	0.1710	0.1490	0.1064
Page-blocks	0.0128	0.0110	0.0142	0.0171	0.0185	0.0161
Optdigits	0.0247	0.0156	0.0280	0.0290	0.0322	0.0227
Mushrooms	0.0081	0.0000	0.0006	0.0013	0.0005	0.0002
Magic	0.0409	0.0284	0.0792	0.0744	0.0818	0.0453
Adult	0.0355	0.0304	0.0640	0.0652	0.0717	0.0196
Shuttle	0.0038	0.0004	0.0008	0.0016	0.0021	0.0004
Connect-4	0.0953	0.0037	0.0883	0.0956	0.1044	0.0294
Waveform	0.0044	0.0009	0.0119	0.0110	0.0102	0.0023
